# Optimisation of Radiation Exposure to Gastroenterologists and Patients during Therapeutic ERCP

**DOI:** 10.1155/2013/587574

**Published:** 2013-03-26

**Authors:** Khalid Alzimami, Abdelmoneim Sulieman, Georgios Paroutoglou, Spiros Potamianos, Marianna Vlychou, Kiki Theodorou

**Affiliations:** ^1^Radiological Sciences Department, College of Applied Medical Sciences, King Saud University, P.O. Box 10219, Riyadh 11433, Saudi Arabia; ^2^Radiology and Medical Imaging Department, College of Applied Medical Sciences, Salman bin Abdulaziz University, P.O. Box 422, Al-kharj 11942, Saudi Arabia; ^3^Public Gastroenterology Department, University Hospital of Larissa, P.O. Box 1425, 41110 Larissa, Greece; ^4^University Gastroenterology Department, University Hospital of Larissa, P.O. Box 1425, 41110 Larissa, Greece; ^5^Radiology Department, University Hospital of Larissa, P.O. Box 1425, 41110 Larissa, Greece; ^6^Medical Physics Department, University Hospital of Larissa, P.O. Box 1425, 41110 Larissa, Greece

## Abstract

This study intended to optimize the radiation doses for gastroenterologists and patients during therapeutic endoscopic retrograde cholangiopancreatography (ERCP) and to compare the doses based on available data obtained by other researchers. A total of 153 patients were studied in two Gastroenterology Departments, (group A, 111; group B, 42). Thermoluminescent dosimeters (TLD) were used to measure the staff and patients entrance surface air kerma (ESAK) at different body sites. The mean ESAK and effective doses per procedure were estimated to be 68.75 mGy and 2.74 mSv, respectively. Staff was exposed to a heterogonous doses. The third examiner (trainee) was exposed to a high dose compared with other examiners because no shield was located to protect him from stray radiation. Patients and examiners doses were lower compared to the lowest values found in previous studies taking into consideration the heterogeneity of patients and equipment. Staff doses during ERCP are within the safety limit in the light of the current practice.

## 1. Introduction

Endoscopic retrograde cholangiopancreatography (ERCP) is considered the gold standard procedure in the evaluation of pancreaticobiliary disorders and the treatment of many clinical conditions with a high successful rate of up to 90% [1]. ERCP has evolved from a diagnostic to an almost exclusively therapeutic procedure after the introduction of new, noninvasive imaging techniques, which provide diagnostic information that allows the selection of patients for therapeutic ERCP [1–3].

However, ERCP requires fluoroscopic and radiographic exposures, which impose radiation risks to patients and examiners. The partial exposure of patient results in a heterogeneous dose distribution; therefore the organ dose and effective dose values are more appropriate descriptors of patient dose and related risks [4].

Moreover, the examiners are required to stand close to the patient in order to conduct the procedure, and for this reason, staff and patient doses are interdependent. As a consequence, unprotected parts by the lead apron (hands, eye lens, and thyroid), may receive significant radiation doses from scattered X-rays [2–9]. Although the radiation dose to examiners is very low, no radiation dose can be considered safe, especially when accumulated under high workload circumstances [2, 3, 8–12]. 

Monitoring of the radiation exposure to patients and examiners is recommended [4]; nevertheless, there are only a few studies published regarding the radiation doses received by the patients, examiners and for both patients and examiners [3, 5–16]. These studies show wide differences in terms of dose, fluoroscopic time, number of radiographic images, equipment, and interexaminers variability, suggesting that patient dose optimisations methods have not been accomplished yet. In addition to that, some studies used phantoms to estimate examiners dose [13, 14]. Even if the above data does not reflect the daily dose management, it is useful in increasing examiners awareness. Furthermore, there is a need of information concerning the doses received by radiosensitive organs, dose optimization, and the related risks. Reference dose levels for ERCP have not yet been adopted either in national or international levels in terms of entrance surface air kerma (ESAK), according to our knowledge. Recently, attempts were made to reduce the dose to patients during ERCP procedures [3].

ESAKs were measured for patients and staff by different methods in literature indirectly with dose area product meter (DAP) (10–13), directly with thermoluminescent dosemeters (TLD) (3, 5, 10, 13, and 14) and with ion chambers (11, 14). All the staff measurements were performed with TLDs, except Oztas et al. [10] who used a personal digital dosimeter.

DAP meters are useful in patient dose measurements of time, while TLD is a reliable dosimeter for ERCP, since the examined area and patient position remained constant throughout the examination [7].

However, TLDs can be used wherever the DAP meter is not available. Furthermore, TLD remains the dosimeter of choice for measurements of staff personal equivalent dose [11, 12]. 

The present investigation aims to (i) measure and compare the radiation dose to patients and examiners in two distinct groups of examiners from two gastroenterology departments. (Department A (Public Gastroenterology Department) and Department B (University Gastroenterology Department)), (ii) compare the doses and reference levels with available literature data, and (iii) evaluate the technique applied in order to reduce patient and examiners doses.

## 2. Materials and Methods

### 2.1. Patient Dose Measurement

One hundred and fifty-three consecutive ERCP procedures were evaluated. The ethics and research committee approved the study, and a written consent was obtained from all patients prior to the procedure. TLDs were packed on a thin envelope made of transparent plastic foil, which contained 3 TLDs, to protect them from any contamination to measure ESAK. During the procedure the TLDs were kept in the required positions and were stuck in place with adhesive tape. The envelopes were positioned accordingly at the center of the field in the case where patient positions had to be changed, especially in the case of postprocedure supine view. The examiners were performing the investigations as their daily practice with a protocol that is designed to minimise patient and examiner doses. Demographic data (age, height, and weight) and exposure factors (kVp and tube current-time product (mAs)) were obtained for all patients.

### 2.2. Examiner Dose Measurement

Two experienced gastroenterologists (>10 years of experience) in group A and group B performed all the procedures, respectively. Regarding the first examiner, the radiation dose was monitored outside the lead apron at 7 sites: the forehead (eye lens), thyroid, chest, left hand, waist (left side), back shoulder at the scapula, and the left leg. The examiner used a 0.25 mm lead equivalent thick apron, full wrap-around protection (a lead apron offering all round protection) (Dr. Goos-Suprema GmbH, Heidelberg, Germany). The second and the third examiners used 0.50 mm lead equivalent thickness, frontal protection (Rheix-srl, Milan, Italy). Gastroenterologists were using different types of lead aprons according to availability. TLD envelopes were attached outside the lead apron at the chest level and at the left hand of the second examiner, while for the third examiner (fellow) the radiation dose was monitored for the hand, chest, thyroid, and forehead. Neither a protective eyeglass nor thyroid collar was worn by either of the examiners. The examiner radiation dose in gastroenterology departments is routinely monitored by TLD dosemeters.

A lead apron (100 × 60 cm^2^) of 0.50 mm lead equivalent was placed on the side of the first examiner over the table to reduce radiation scatter to the examiners standing to the side of the fluoroscopy couch. 

The second examiner (assistant) also controlled the radiation exposure from inside the room, and the third examiner stood near the patient head for training purposes in group B. All procedures were performed with the examiners at the same locations. The nurses remained outside the X-ray room during the exposure. 

**Figure 1:**
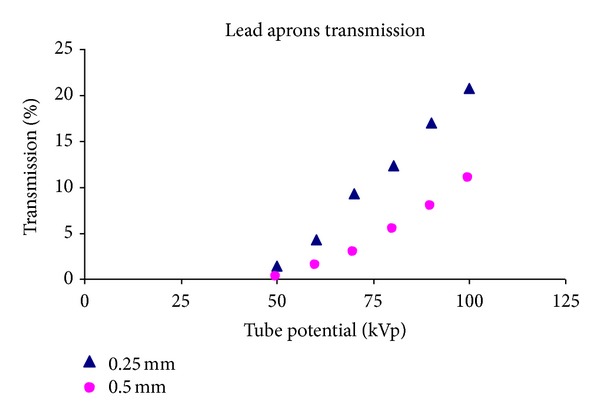
The transmission ratio versus the tube potential (kVp).

The transmission of 0.25 mm and 0.50 mm lead equivalent aprons was measured with different radiation qualities ranging from 50 kVp to 100 kVp. The aprons were placed in the primary beam, and the entrance and exit doses were measured at source surface distance (SSD) of 1 meter using the ionisation chamber (Figure 1). In cases of thicker lead apron (0.5 mm) accompanied by 75 kVp, *H*_U_ for the thicker apron is more like 5% of *H*_OS_ rather than 10% as illustrated in Figure 1.

### 2.3. ERCP Technique

All cases were performed for therapeutic purposes. ERCP was performed with a duodenoscope (Olympus, Exera CLE 145 (Olympus Medical System Corp, Japan)). The patient was placed on an X-ray couch in the left anterior oblique position. During the procedure, radiographic and fluoroscopic images were obtained after injection of contrast medium. Radiographic exposures were made using digital fluorography. Since the contrast medium normally remained in the biliary tree for several minutes following removal of the duodenoscope, a postprocedure anteroposterior projection was also obtained, if required, for further evaluation of the stent placement or residual stones.

### 2.4. Dose Optimisation Protocol

The study protocol was designed to use intermittent fluoroscopy (low fluoroscopic time), use fluoroscopic images, and reduce number of radiographic films. Patients were precisely positioned and focused prior imaging to eliminate unnecessary radiation exposure. The quality of the fluoroscopic image was acceptable. However, in some cases, that were not considered diagnostically acceptable, additional radiographic images were acquired. Radiographic images were taken in cases of difficulty in evaluating a finding.

### 2.5. TLD Measurements

Radiation dose measurements were made using two groups of TL dosemeters from Harshaw TLD (Bicron-NE, Solon, OH, USA) of dimensions 3.1 × 3.1 × 0.89 mm^3^. TLD-100 was selected to measure patient doses, while TLD-200 was selected for examiner dose measurements for their numerous advantages [17]. 

TLD calibration was according to international protocols for the range of energies used in the study [18]. The TLDs were calibrated under reproducible reference conditions using the same X-ray machine used to perform ERCP procedure (Philips Diagnost 93) against an ionization chamber model 9060/10X5-60 connected to a Radiation Monitor Controller model 9010 (Radcal Corporation, Monrovia, CA). Both the chamber and the electrometer were calibrated for the energy ranges 30–120 kV at the National Standard Laboratory. For the TLD and chamber irradiation, a polymethylmethacrylate (PMMA) calibration test bed has been constructed having dimensions 30 × 30 × 10 cm^3^, which simulates the patient's lateral and backscatter conditions. The first PMMA slab was used to accommodate the TLD chips in an array of slots 10 × 10. Each TLD was identified by its position in the array. Individual calibration factors were obtained by irradiating the entire group to the same dose. The measured signal of each TLD was divided by the mean signal of the group. This process was repeated three times to reduce the effect of statistical variations and to determine the stability and reproducibility of the signal. TLDs with sensitivity within 3% were used in this study. All the TLD chips had the same thermal history. The calibration cycle was carried out every month.

The TLD signal was read using a manual TLD reader (Harshaw 3500, Solon, USA).

The readout was at a 100°C preheat temperature and reading temperature of 100–280°C with heating rate 10°C s^−1^. Before each irradiation all dosimeters were annealed in an annealing oven (TLDO; PTW, Freiburg, Germany) at 400°C for 1 h followed by fan-forced cooling down to 100°C which was held for 2 h. Postirradiation annealing was carried out for 10 min at 100°C.

Four TLDs were irradiated to a known dose (standard dose) of the same radiation quality under the standard conditions. A second group of four TLDs was also irradiated to another known dose (test dose) to check the accuracy of the standard dose. The radiation dose received by the patient was calculated using the individual calibration factors of the chips and relating that reading to the reading of the standard TLDs, which had received a known dose. The mean background signal for unirradiated TLDs was subtracted before any calculation. The linearity of the TLD's response for the range of dose used in this study had been verified. The uncertainty of TLD reading was estimated to be not more than ±9% according to the test dose results.

### 2.6. Radiographic Equipment

ERCP procedures were performed using an overcouch machine (Philips Diagnost 93). The filtration of the X-ray beam was 4.0 mm Al. The kVp and mAs ranges are 40–125 and 1–850, respectively. Last image hold capability, automatic brightness control, and footswitch are available. The machine had three fields of views: 17, 25, and 31 cm and an audible alarm system preset to sound at 5 minutes intervals, which alerted the team that fluoroscopic time has elapsed. The nominal focus to image intensifier distance (FID) used was fixed at 110 cm. The resolution of the image intensifier is 1.8 lp mm^−1^ 21, and the geometrical distortion is 6% at the centre and 11% at the periphery, resulting in very good image quality. During the procedure, tube potential setting is set manually, mAs being under automatic exposure control.

### 2.7. Estimation of Patient-Absorbed Organ Doses and Effective Doses

ESAK was used to estimate the organ equivalent dose (H) using software provided by the National Radiological Protection Board (NRPB-SR262) [19]. 

However, as specific projections were not available for ERCP, effective dose was obtained from the average value of the conversion factors for the most similar PA kidney, stomach, and oblique duodenum views.

## 3. Results 

A total of 153 were investigated. The mean age for all patients was 65.3 years which ranged from 26 years to 90 years as presented in Table 1. The mean and range of BMI of the patients were 74.9 (47–106) and 73.5 (50–110) for group A and B, respectively. The mean exposure factors, number of films, and fluoroscopic time (min), for both groups, are shown in Table 2. The patient exposure factors are higher in group B compared to group A. The measured patients doses in terms of ESAK (mGy) values for both patients groups are shown in Table 3. The results of patient dose show asymmetry in the dose distribution. Therefore, the parameter of central tendency is preferred. The ESAK for group A is 15% lower than group B. This difference may partly be due type of procedure and interexaminer differences, since the examiners were using the same X-ray machine. The examiners doses (*µ*Gy) per procedure for both groups are presented in Table 4. The dose values received by both groups are comparable. Tables 5 and 6 presented the results of dose value for the second and third examiners, respectively. The third examiner exposed to higher doses in all monitored locations compared to the two examiners.

## 4. Discussion

Medical radiation exposures impose risks to patients and staff. Therefore, it is imperative to measure the dose received by the staff to evaluate whether they are within the prescribed annual dose limits to reduce the probability of stochastic effect. Regarding the increasing number of patients undergoing ERCP procedures, particular attention to radiation protection for patients and staff is required. In general, radiation dose is optimized when imaging is performed with the least amount of radiation required to provide adequate image quality and imaging guidance. Radiation management requires that factors is to be considered and that steps are to be taken during ERCP procedure [14]. The mean ESAK and E resulting from ERCP procedure has been quantified to be 68.89 mGy and 2.75 mSv, respectively, for the total patient population. The mean ESAK for group A was 65.75 mGy, whereas the mean measured value for group B was 77.4 mGy (Table 2). 

The study protocol was designed to use intermittent fluoroscopy with fluoroscopic image with last image hold to minimize exposures. Even if these images generally have inferior image quality compared to radiography, it has the required findings. In this study two experienced gastroenterologist performed all the procedures in group A, and two gastroenterologists and a fellow for training purposes performed all the procedures in group B. Therefore, the presence of two gastroenterologists enabled them to reduce patient doses, and this enabled them to apply these optimization techniques. A similar results were reported by Buls et al [14]. 

 This reduction offers further margins for further investigations and followsup especially for young and pregnant patients. Furthermore, no side effects that were reported during this study could be attributed to radiation dose optimisation protocol. It is important to note that understanding the limitations of equipment while limiting radiation delivery from the specific equipment used in the procedure is an important goal to keep risk low of probability of CBD stone missing. 

**Figure 2:**
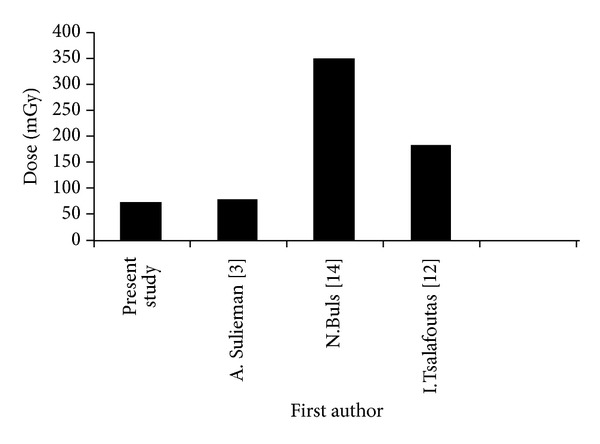
Patient ESD (mGy) compared with previous ERCP studies.

 The results of patient doses in this study are lower compared to previous studies taking into consideration the heterogeneity of patients and equipment (Figure 2). The patient doses in the literature were ranged between 347 mGy per procedure in study published in 2002 [14]. The patient doses in recent published studies were lower compared with the old ones. This might be attributed to the advancement in radiological equipment and staff awareness. 

In this study, staff doses were monitored using extra TLD envelopes for different body locations in order to measure doses per procedure. The measured examiners ESAK for both groups are comparable and presented in Tables 4, 5, and 6 for first, second, and third examiners, respectively. As expected, the first examiner was more exposed than the second one, while the radiation dose for the fellow was the highest because no protective shield was used in that direction, and he was always facing the primary beam. The back shoulder and the waist were points of the highest dose for the first examiner, because these parts are always facing the source of the scatter radiation.

The use of full wrap-around apron is more effective due to the full coverage of the back and waist areas than the frontal protection one. The mean and the range for waist and shoulder doses are higher than examiner's chest and thyroid doses because the examiners are always focusing on the fluoroscopic monitor during the screening. Therefore, the current effective dose estimation method is underestimating the dose, since the TLD should be placed on areas of the body with the highest exposure rate [7, 20]. Also in cases of partial body exposures the reading of a personal dosemeter may not provide a representative value for the assessment of effective dose.

In contrast, many examiners do face the primary beam when they look at ceiling-mounted fluoroscopy and endoscopy screens situated on the far side of the screening table. It is clearly considered that the radiation dose inside the lead apron is insignificant and the examiners are adequately protected, in the light of the fact that the transmissions of the lead apron (0.25 mm and 0.50 mm lead equivalent) are 12.6% and 5% at 80 kVp, respectively, which was determined experimentally (Figure 1).

In agreement with Johlin et al. [16], the third examiner receives the highest dose because he always facing the radiation source. In contrast with Oztas et al. [10], the first examiner received a higher dose than the first one. Buls et al. [14] reported that the mean second examiner (nurse) doses are 260 *µ*Gy, 200 *µ*Gy, and 270 *µ*Gy for eye, neck (thyroid), and hand, respectively (Table 7). The third examiner dose had a dose 25% higher than the second one. In a phantom study, Johlin et al. [16] estimated the dose to examiners during 1 hour of fluoroscopy at 1 meter. The dose to the first, second, and third examiner was 13 *µ*Gy, 12 *µ*Gy, and 270 *µ*Gy, respectively. A reduction of dose up 95% was obtained with rubberized 0.50 mm equivalent lead shield. Table 7 shows first gastroenterologist radiation dose in comparison with previous studies. The doses showed wide variations. Dose differences to examiners can be explained in the light of patient dose differences, examiners location, and the utility of radiation barriers X-ray tube position (over/under couch). The use of overcouch X-ray tube increases the scatter dose to the face, neck, and the upper parts of the body, while undercouch X-ray tube has higher exposure to the legs and lower parts of abdomen depending on the distance to the patient's irradiated volume. While working conditions may be easier with overcouch X-ray machine, the potential exposure of workers is from two to three times greater compared with the undercouch X-ray machines. If an overcouch tube/undercouch intensifier system is not operated by remote control, additional protective drapes shall be provided to ensure adequate protection of the operator [5, 7]. In addition to that, staff experience positively affects the fluoroscopic time and patient dose.

Radiation dose reduction can be achieved by wearing the protective eyeglasses and thyroid shields that significantly attenuate scatter radiation. However, the examiners believe that the use of lead glasses impairs vision and so increases the exposure time and that lead gloves are inconvenient for the first and the second examiners [3]. In this study, the application intermittent fluoroscopy with fluoroscopic image with last image, storing fluoroscopic images, number of radiographic films reduction, reduction of fluoroscopic time. Significant radiation dose reduction can be achieved by applying the factors, which are mentioned in Table 8 in the literature. However, examiners cannot control some variables, such as patient size, procedure type, or fluoroscopic equipment used [7, 21].

In light of the results of this study, the third examiner was moved from the specific place, and all examiners wore wrap-around lead protection with thyroid shields. 

## 5. Conclusion

This study underlines the importance of the protection in busy gastroenterology departments. The unnecessary radiation exposure can be reduced significantly by the presence of two experienced examiners. The radiation dose to the examiners is well within established safety limits, in the light of the current practice. Radiation exposure is determined by patient, procedure, equipment, and staff experience. Furthermore, the first examiner should put on a lead wrap-around protective apron, since he is not facing the scattered radiation. We believe that the available formulae to evaluate effective dose to examiners underestimate the effective dose for ERCP examiners. The results encourage examiners for further dose optimisation. Additional studies need to be conducted in order to establish reference dose levels to patients and effective dose estimation to examiners during ERCP.

## Figures and Tables

**Table 1:** ERCP clinical indications.

Indications	Group A	Group B	Total	%
CBD stones	66	20	86	56.2
Postoperation leakage	5	1	6	3.90
Cholangitis	4	7	11	7.2
Malignant tumors	22	6	28	18.3
Benign CBD stricture	1	3	4	2.6
Stent removal or exchange	4	2	6	3.9
Others	9	3	12	7.8

Total	111	42	153	100

**Table 2:** The mean and range of the exposure parameters for radiography and fluoroscopy for both patients groups.

Group	*n*	Radiography		Fluoroscopy		Number. of radiographic images	Procedure duration (minutes)
	153	Tube voltage (kV)	Tube current-time product (mAs)	Tube voltage (kV)	Tube current-time product (mAs)	2.6 (1–6)	27 (15–55)
A	111	84 (70–100)	44 (12–141)	75 (52–110)	1.6 (0.4–3.1)	2.3 (1–5)	25 (15–50)
B	42	80 (70–104)	58 (10–171)	75 (55–110)	2.2 (1.3–3.1)	3.8 (1–6)	30 (20–55)

**Table 3:** Minimum, median, mean, third quartile, and maximum values of ESAK, exit air kerma, and thyroid entrance air kerma for all patients and for groups A and B separately.

Patient group	*n*	Mean	Minimum	Median	3rd quartile	Maximum
ESAK: all	153	68.75	10.17	44.79	86.81	289.1
Group A	111	65.89	10.17	36.77	74.59	277.1
Group B	42	77.4	14.44	59.41	88.81	289.1

**Table 4:** First gastroenterologist mean radiation doses (*μ*Gy) for groups A and B per ERCP procedure. The range is in parenthesis.

First gastroenterologist	Group	*n*	Radiation dose (*μ*Gy)
Chest	All	153	6.4 (0.2–35.1)
	A	111	6.1 (0.2–37.8)
	B	42	6.7 (1.1–30.1)
Thyroid	All	153	5.40 (0.02–27.6)
	A	111	5.40 (0.2–27.6)
	B	42	5.52 (1.03–26.1)
Eye lens	All	153	3.81 (0.2–26.3)
	A	111	3.51 (0.2–26.3)
	B	42	4.45 (0.7–21.0)
Hand	All	153	27.2 (1.02–223.2)
	A	111	27.3 (1.02–223.2)
	B	42	26.9 (2.9–171.5)
Back shoulder	All	85	38.7 (0.70–282.3)
	A	61	60.1 (0.70-282.3)
	B	36	39.3 (1.25–191.2)
Waist	All	85	100.5 (13.6–381.3)
	A	61	101.2 (13.6–381.3)
	B	36	99.5 (14.7–291.0)
Leg	All	54	1.60 (0.2–17.1)
	A	30	1.50 (0.2–15.9)
	B	24	2.00 (0.2–17.1)

*n*: sample size.

**Table 5:** Second gastroenterologist mean radiation doses (*μ*Gy) for groups A and B per ERCP procedure. The range is in parenthesis.

Second gastroenterologist	Group	*n*	Mean
Chest	All	153	5.7 (0.2–25.4)
	A	111	4.8 (0.2–23.1)
	B	42	6.4 (1.1–25.4)
Hand	All	153	7.2
	A	111	8.4
	B	42	4.3

**Table 6:** Third staff (trainee) mean radiation doses (*μ*Gy) for group B per ERCP procedure. The range is in parenthesis.

Third examiner	*n*	Mean
Chest	24	72 (12.8–429)
Thyroid	24	63 (16.1–382.1)
Forehead	24	65 (13.4–317.8)
Hand	24	162 (32.1–739.5)

*n*: sample size.

**Table 7:** The mean radiation doses for the first examiner during ERCP procedure (*μ*Gy).

First examiner	Hand	Thyroid	Eye lens	Chest	No. of Film	Exposure time (min)	X-ray tube location
Present study	27.0	5.0	3.0	6.0	2.5	2.4	Overcouch
Buls et al [14]	0.64	0.45	0.55	nd	4	6.0	Overcouch
Naidu et al. [9]*	NR	0.2	0.04	NR	4.6	5.9	Overcouch
Sulieman et al. [3]	27.2	5.4	3.8	6.2	2.6	2.9	Overcouch
Oztas et al. [10]	20.0	2.0	82.0	NR	1.7	5.7	undercouch

*Extrapolated from annual effective dose (mSv) for 400 procedures per year.

NR: not reported.

**Table 8:** Patients dose reduction techniques during ERCP.

Imaging parameters	Equipment settings characteristics	ERCP procedure

Increase tube voltage	Reduce image intensifier patient distance	Experienced examiners
Reduce fluoroscopic time	Undercouch configuration	Well patient positioning and focusing prior the procedure
Reduce number of radiographic films	Fluoroscopy time with alarm	Radiation barriers
Intermittent fluoroscopy	Last image hold with digital features	Wear wrap-around lead aprons
Storing fluoroscopic images	Adequate filtration	Examiners radiation safety training
Selection of the low-dose fluoroscopic mode	Pulsed fluoroscopy	Examiners and patient dose monitoring
Avoid magnification	Radiation control from inside the room	Dose reference levels
Radiation field collimation	Automatic brightness control (ABC)	Thyroid shields
Pulsed mode fluoroscopy	Mobile/suspended screen	ALARA principles (as low as reasonably achievable)
